# Isolated tumor cells in stage I & II colon cancer patients are associated with significantly worse disease-free and overall survival

**DOI:** 10.1186/s12885-016-2130-7

**Published:** 2016-02-16

**Authors:** B. Weixler, R. Warschkow, U. Güller, A. Zettl, U. von Holzen, B. M. Schmied, M. Zuber

**Affiliations:** Department of Surgery, Kantonsspital Olten, Baslerstrasse 150, CH - 4600 Olten, Switzerland; Department of Surgery, Kantonsspital St. Gallen, St. Gallen, Switzerland; Department of Oncology/Hematology, Kantonsspital St. Gallen, St. Gallen, Switzerland; University Clinic for Visceral Surgery and Medicine, Inselspital Berne, University of Berne, Berne, Switzerland; Viollier AG, Histopathology/Cytology, Basel, Switzerland; Department of Surgery, University Hospital Basel, Basel, Switzerland; Institute of Medical Biometry and Informatics, University of Heidelberg, Heidelberg, Germany

**Keywords:** Isolated tumor cells, Colon cancer, Survival

## Abstract

**Background:**

Lymph node (LN) involvement represents the strongest prognostic factor in colon cancer patients. The objective of this prospective study was to assess the prognostic impact of isolated tumor cells (ITC, defined as cell deposits ≤ 0.2 mm) in loco-regional LN of stage I & II colon cancer patients.

**Methods:**

Seventy-four stage I & II colon cancer patients were prospectively enrolled in the present study. LN at high risk of harboring ITC were identified via an in vivo sentinel lymph node procedure and analyzed with multilevel sectioning, conventional H&E and immunohistochemical CK-19 staining. The impact of ITC on survival was assessed using Cox regression analyses.

**Results:**

Median follow-up was 4.6 years. ITC were detected in locoregional lymph nodes of 23 patients (31.1 %). The presence of ITC was associated with a significantly worse disease-free survival (hazard ratio = 4.73, *p* = 0.005). Similarly, ITC were associated with significantly worse overall survival (hazard ratio = 3.50, *p* = 0.043).

**Conclusions:**

This study provides compelling evidence that ITC in stage I & II colon cancer patients are associated with significantly worse disease-free and overall survival. Based on these data, the presence of ITC should be classified as a high risk factor in stage I & II colon cancer patients who might benefit from adjuvant chemotherapy.

## Background

Colon cancer still remains one of the leading causes of cancer related death and represents a tremendous public health problem. The TNM staging system discriminates nodal negative (stage I & II) from nodal positive (stage III) disease. Adjuvant treatment is usually reserved to stage III disease. It is assumed that complete surgical resection can be achieved in stage I & II colon cancer and therefore no further treatment is recommended for most of these patients. Unfortunately, up to 20 % of stage I & II disease patients will develop recurrence within five years after diagnosis. The cause of this high recurrence rate remains unclear [1, 2]. However, the identification of factors predicting a worse survival in stage II colon cancer led the American Society of Clinical Oncology (ASCO) [3–5], the Nationonal Comprehensive Cancer Network (NCCN) and the European Society for Medical Oncology (ESMO) to define a collective of high-risk patients who may benefit from adjuvant chemotherapy. Those high-risk stage II colon cancer patients feature at least one of the follwing characteristics: pT4 tumor, poorly differentiated histology, presence of lymphovascular invasion, localized perforation, bowel obstruction or less than 12 lymph nodes (LN) analyzed [4, 6]. The patient benefit of an adjuvant treatment in this subgroup, however, remains a matter of debate [7].

While uncertainty persists regarding the explanation of the high recurrence-rate in node negative colon cancer, there is emerging evidence that the appearance of isolated tumor cells (ITC) and micro-metastases in LN could be associated with worse prognosis [8–14]. According to the TNM classification system micro-metastases are defined as tumor deposits of 0.2 mm to ≤ 2 mm in diameter, labeled as pN1(mi), and ITC as either single tumor cells or clusters of tumor cells of 0.2 mm or less, labeled as pN0(i+) [15].

The worldwide standard of histopathologic analysis of colon cancer LN represents a single-level sectioning and hematoxylin-eosin (H&E) staining through each discovered LN. However, this method provides a very limited access to the examined tissue and implies a relevant risk of sampling bias and understaging [16]. Indeed, the detection of ITC usually requires either molecular methods [17] or step sectioning combined with immunohistochemistry and hence, ITC are often missed using standard H&E staining. Therefore, a few research groups have been evaluating the sentinel lymph node (SLN) procedure in colon cancer to allow a more thorough investigation of a few LN with high probability of hiding tumor infiltrates. SLN assessment has been reported to lead to an upstaging of up to 15 % of patients with initially node negative colon cancer [16, 18–20].

There is rising evidence that colon cancer patients with micro-metastases will have a prognosis similar to patients with macro-metastases but little has been published about the prognostic impact of ITC. Furthermore, the vast majority of published studies are either retrospective or do not differentiate between colon and rectal cancer [8, 21, 22]. To date only seven studies exist which investigate the influence of ITC in node negative colon cancer [12].

Therefore, the objective of our prospective study was to asses the prognostic impact of ITC on disease-free and overall survival in stage I & II colon cancer patients.

## Methods

This study was performed between January 2005 and December 2012 in an university affiliated hospital (Kantonsspital Olten) and was designed as a prospective single center trial. The study was approved by the ethics committee EKNZ (Ethikkommission Nordwest- und Zentralschweiz) and all patients had given written informed consent prior to surgery.

Patients undergoing primary resection for histologically proven colon cancer were admitted to open surgery and - after providing written informed consent - analyzed according to the Swiss SLN protocol. This procedure consists of the in-vivo peritumoral injection of isosulfan blue to identify the SLN, a procedure described in detail elsewhere [19]. Only node negative colon cancer patients, i.e. pN0 (stage I & II) were included in this study. A total of 74 patients could be analyzed. Patients were divided into two groups, patients in whom ITC were detected and patients who were truly node negative.

### Histopathologic examination

Five serial sections were obtained at 3 different levels of each marked SLN. H&E staining was then performed for the first section of each level. If no metastatic deposits were detected, an immunohistochemical staining with AE1/AE3 or CK19 was conducted for the fourth section of each level. To collect the remaining non-SLN, the fixed specimen was then manually dissected. Bivalving and H&E staining of the non-SLN were then carried out. All cytokeratin positive cells were confirmed to be tumor cells, based on morphological characteristics, by microscopic reevaluation of the immunostained sections after counterstaining with hemalaun.

### Staging

After completion of the study in December 2012, all histopathologic reports were reviewed and staging was then performed according to the 7th edition of the UICC staging manual [23]. Isolated tumor cell deposits ≤ 0.2 mm were considered as ITC [15, 24]. Patients in whom ITC were detected after the above described histopathological in-depth analyses were staged as pN0(i+).

### Data collection and definitions

Survival and disease recurrence data were obtained by phone interview with the responsible oncologist and/or general practitioner. Adjuvant chemotherapy was given according to interdisciplinary tumor board decisions. This decision was made individually for each patient and was based on the presence of high risk factors (T4, lymphovascular invasion, poor differentiation, etc.) and the patient’s general condition. ITC were not considered as an indication for adjuvant chemotherapy.

### Statistical analyses

Statistical analyses were performed using the R statistical software (www.r-project.org). A two-sided *p*-value < 0.05 was considered statistically significant. Continuous data are expressed as means ± standard deviations. For comparing proportions, Chi-Square statistics and for comparing continuous variable t-tests were used. Disease-free survival was defined as the primary outcome variable.

First, the risk for ITC was assessed regarding age, gender, tumor localization, tumor staging, grading, lymphovascular invasion, preoperative CEA levels, number of extracted lymph nodes, number of sentinel lymph nodes, and adjuvant therapy. The same set of covariates including ITC were then assessed as putative prognostic factors for disease-free and overall survival in unadjusted and risk-adjusted Cox regressions including a backward variable selection procedure from the full Cox regression model based on the Akaike’s information criterion.

The relative survival – as a validated mean to reflect the cancer-specific survival – was estimated [25]. Relative survival was calculated as the ratio of the observed overall survival rate and the expected population-based survival rate (“background mortality”) [26]. The population tables regarding background mortality for the relative survival analyses were obtained from the Swiss National Statistical Office [27]. The relative survival analyses were conducted using the R package “relsurv” using the Pohar-Perme-estimator [28]. Population mortality rates were included as time-dependent covariates in multiplicative Cox regression model [29].

## Results

### Patient characteristics and propensity for detection of ITC

A total of 74 patients with a median follow-up time of 4.6 years (range:1 month to 8.0 years) were eligible for the present analysis. On average, 28.5 ± 11.7 LN were resected with an average of 5.8 ± 3.4 SLN. In 23 of the 74 stage I & II patients (31.1 %) ITC were detected. Table 1 summarizes the patient characteristics and the outcomes. Differences between patients with and without ITC did not reach the significance level except the use of adjuvant therapy (Table 1). In univariate and multivariable logistic regression, adjuvant therapy again was the only variable independently associated with the detection of ITC (odds ratio = 5.65, 95 % CI = 1.34 to 29.2). The nine patients who received adjuvant therapy did have stage II colon cancer and all of them featured at least one high risk factor. These nine patients were younger (62.8 ± 13.8 years vs. 71.7 ± 10.7 years, *p* = 0.093), had more advanced tumor stages (AJCC stage II in 9 out of 9 patients vs. 37 of 65 patients, *p* = 0.010), had more often a lymphovascular invasion (4 of 9 patients vs. 7 of 65 patients, *p* = 0.026), and had more frequently elevated preoperative CEA levels (4 of 9 patients vs. 11 of 65 patients, *p* = 0.090).

**Table 1:** Patient characteristics and outcome for stage I & II colon cancer patients

Patient characteristics		Total *N* = 74	ITC *N* = 23	No ITC *N* = 51	*p*
Age	(Years)	70.6 ± 11.4	69.3 ± 10.2	71.2 ± 11.9	0.494 A)
Gender	Male	39 (52.7 %)	13 (56.5 %)	26 (51.0 %)	0.659 B)
	Female	35 (47.3 %)	10 (43.5 %)	25 (49.0 %)	
Tumor localisation	Colon caecum	12 (16.2 %)	1 (4.3 %)	11 (21.6 %)	0.084 B)
	Ascending colon	16 (21.6 %)	5 (21.7 %)	11 (21.6 %)	
	Transverse colon	13 (17.6 %)	4 (17.4 %)	9 (17.6 %)	
	Descending colon	7 (9.5 %)	5 (21.7 %)	2 (3.9 %)	
	Rectosigmoid colon	26 (35.1 %)	8 (34.8 %)	18 (35.3 %)	
T-stage	I	7 (9.5 %)	0 (0.0 %)	7 (13.7 %)	0.264 B)
	II	21 (28.4 %)	7 (30.4 %)	14 (27.5 %)	
	III	42 (56.8 %)	14 (60.9 %)	28 (54.9 %)	
	IV	4 (5.4 %)	2 (8.7 %)	2 (3.9 %)	
Grading	I	1 (1.4 %)	0 (0.0 %)	1 (2.0 %)	0.792 B)
	II	47 (63.5 %)	15 (65.2 %)	32 (62.7 %)	
	III	26 (35.1 %)	8 (34.8 %)	18 (35.3 %)	
Lymphovascular	No	63 (85.1 %)	17 (73.9 %)	46 (90.2 %)	0.091 C)
invasion	Yes	11 (14.9 %)	6 (26.1 %)	5 ( 9.8 %)	
Preoperative CEA	<5 μg/l	59 (79.7 %)	16 (69.6 %)	43 (84.3 %)	0.167 C)
levels	≥5 μg/l	15 (20.3 %)	7 (30.4 %)	8 (15.7 %)	
TotalLNn	(n)	28.6 ± 11.7	27.7 ± 11.5	29.0 ± 11.9	0.668 A)
SLNn	(n)	5.8 ± 3.4	6.0 ± 3.5	5.6 ± 3.4	0.685 A)
NSLNn	(n)	22.9 ± 11.6	21.7 ± 10.6	23.4 ± 12.1	0.565 A)
Adjuvant therapy	No	65 (87.8 %)	17 (73.9 %)	48 (94.1 %)	0.025 C)
	Yes	9 (12.2 %)	6 (26.1 %)	3 (5.9 %)	
Recurrence	No	71 ( 95.9 %)	21 (91.3 %)	50 ( 98.0 %)	0.254 C)
	Yes	3 (4.1 %)	2 (8.7 %)	1 (2.0 %)	
	Yes: liver	1 (1.4 %)	0 (0.0 %)	1 (2.0 %)	
	Yes: lung	2 (2.7 %)	2 (8.7 %)	0 (0.0 %)	
Death	No	58 (78.4 %)	16 (69.6 %)	42 (82.4 %)	0.239 C)
	Yes	16 (21.6 %)	7 (30.4 %)	9 (17.6 %)	

*n* (%); mean ± standard deviation

A) t-Test, B) Chi-squared test, C) Mid-p test

### ITC as a prognostic factor for disease-free survival

**Fig. 1:**
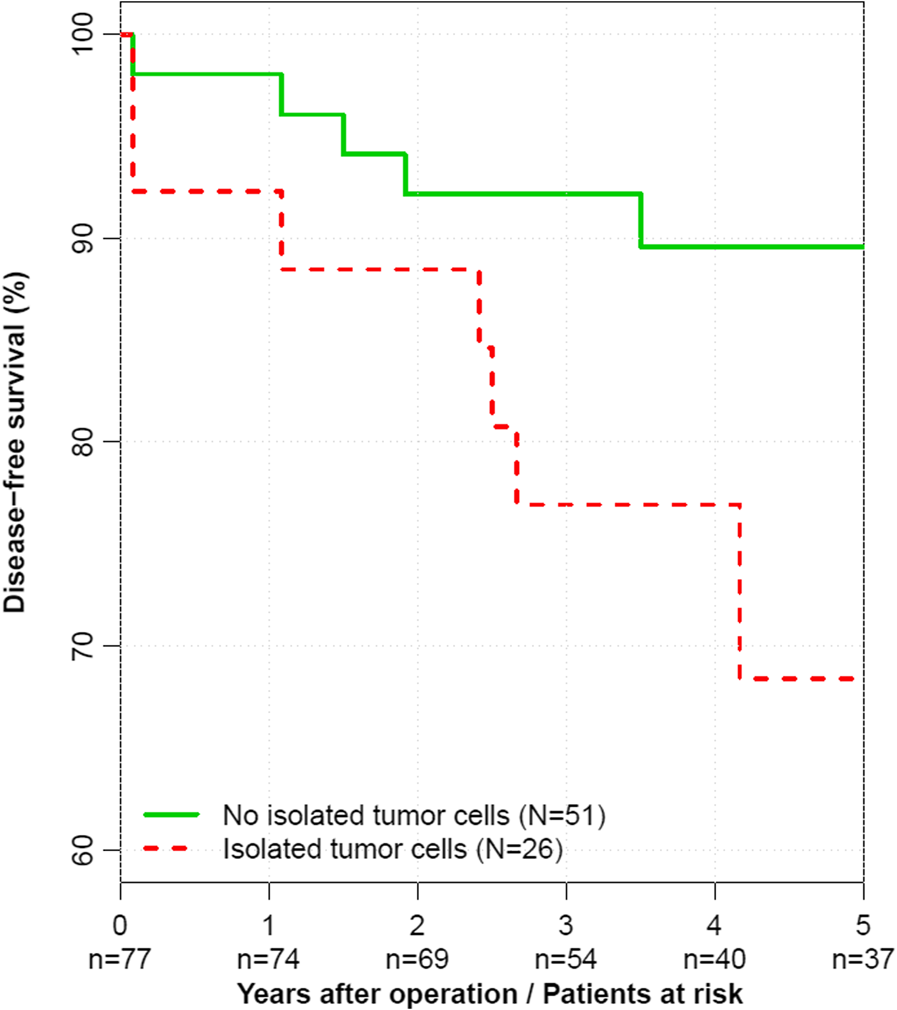
Kaplan–Meier curve for disease-free survival in unadjusted analysis. The number of colon cancer patients at risk are given below each plot

An unadjusted Cox proportional hazards regression analysis revealed ITC as a prognostic factor for disease-free survival with an approximately 182 % increased risk for recrurrence (hazard ratio (HR) of death = 2.82, 95 % CI = 1.06 to 7.49, *p* = 0.043) (Table 2). The five-year disease-free survival for patients with ITC was 63.4 % (95 % CI = 43.0 % to 93.4 %) compared to 89.6 % (95 % CI = 81.3 % to 98.7 %) in patients without ITC (Fig. 1). After adjusting for potential confounders in multivariable Cox regression analyses, ITC remained associated with a worse disease-free survival (HR = 3.69, 95 % CI = 1.18 to 10.9, *p* = 0.024). Moreover, a backward variable selection procedure revealed ITC as an independent factor for higher risk of recurrence (HR = 4.73, 95 % CI = 1.67 to 13.4, *p* = 0.005).

**Table 2:** Prognostic factors for disease-free survival after colon cancer resection

Prognostic factors		Cox Regression					
		Unadjusted ^A)^		Full model ^B)^		Variable selection ^C)^	
		HR (95 % CI)	*p* ^D)^	HR (95 % CI)	*p* ^D)^	HR (95 % CI)	*p* ^D)^
ITC	No	Reference	0.043	Reference	0.024	Reference	0.005
	Yes	2.82 (1.06–7.49)		3.59 (1.18–10.9)		4.73 (1.67–13.4)	
Age	(years)	1.10 (1.03–1.17)	0.001	1.12 (1.03–1.22)	<0.001	1.13 (1.05–1.21)	<0.001
Gender	male	Reference	0.260	Reference	0.220	-	-
	female	0.57 (0.21–1.55)		0.51 (0.17–1.53)		-	-
Tumor localisation	colonic	Reference	0.428	Reference	0.886	-	-
	rectosigmoidal	0.64 (0.21–2.00)		0.91 (0.24–3.47)		-	-
AJCC stage	I	Reference	0.569	Reference	0.581	-	-
	II	1.35 (0.47–3.84)		1.39 (0.43–4.52)		-	-
Grading	GI/GII	Reference	0.356	Reference	0.952	-	-
	GIII	1.62 (0.57–4.62)		1.04 (0.28–3.84)		-	-
Lymphovascular	No	Reference	0.182	Reference	0.368	-	-
invasion	Yes	2.30 (0.74–7.12)		2.04 (0.45–9.31)		-	-
Preoperative CEA	<5 μg/l	Reference	0.304	Reference	0.302	-	-
levels	≥5 μg/l	0.49 (0.11–2.15)		0.42 (0.07–2.44)		-	-
TotalLNn	(n)	0.98 (0.94–1.02)	0.323	1.00 (0.95–1.05)	0.898	-	-
SLNn	(n)	1.00 (0.86–1.15)	0.954	1.02 (0.85–1.22)	0.850	-	-
Adjuvant	No	Reference	0.976	Reference	0.705	-	-
Chemotherapy	Yes	0.98 (0.22–4.33)		1.47 (0.21–10.4)		-	-

HR - Hazard ratios with 95 % confidence intervals (Wald type)

Prognostic factors for overall survival in

A) one Cox proportional hazards regression analyses for each factor

B) Cox proportional hazards regression analyses for all factors

C) Cox proportional hazards regression analyses after backwards variable selection

D) *p* values for likelihood ratio test

### ITC as a prognostic factor for overall survival

**Fig. 2:**
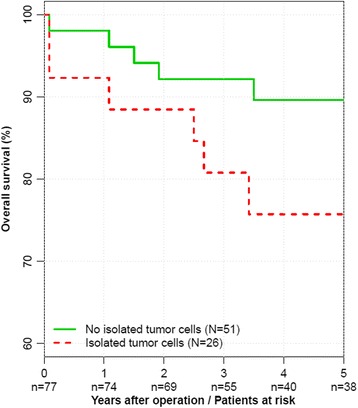
Kaplan–Meier curve for overall survival in unadjusted analysis

Unadjusted Cox proportional hazards regression analysis did not reveal ITC as a statistically significant prognostic factor for overall survival (HR of death = 2.27, 95 % CI = 0.83 to 6.17, *p* = 0.119). ITC were an independent significant prognostic factor for overall survival after risk-adjusting in multivariable Cox regression analysis (HR of death = 3.50, 95 % CI = 1.03 to 11.8, *p* = 0.043) and in backward variable selection (hazard ratio of death = 4.48, 95 % CI = 1.50 to 13.4, *p* = 0.010) (Fig. 2).

### ITC as a prognostic factor for relative survival

A Cox proportional hazards regression analysis with adjustment for the population-based background mortality revealed ITC as a significant poor prognostic factor for relative survival (hazard ratio of death = 3.31, 95 % CI = 1.22 to 9.03, *p =* 0.025). When further adjusting for other confounders in multivariable analysis, ITC remained a significant poor prognostic factor for relative survival (hazard ratio of death = 3.83, 95 % CI = 1.12 to 13.1, *p =* 0.032). For patients without ITC, the relative survival did not significantly exceed the survival one would expect for the Swiss population matched for age, gender and year of operation (*p =* 0.950). However, for patients with ITC, the survival was significantly worse when comparing with the Swiss population matched for age, gender and year of operation (*p =* 0.001). For patients without ITC, nine deaths were recorded (9/51, 17.6 %), three occurred due to tumor recurrence (3/51, 5.9 %). For patients with ITC, seven deaths were recorded (7/23, 30.4 %), two occurred because of tumor recurrence (2/23, 8.7 %).

## Discussion

The present study provides compelling evidence that ITC have a significant negative impact on both disease-free and overall survival in stage I&II colon cancer patients.

LN involvement represents the most important prognostic factor in colon cancer patients [24, 30, 31]. Despite complete surgical resection and the call for a minimum of 12 analyzed LN for adequate staging, an unsettling high recurrence rate in stage I & II colon cancer patients remains. Based on the present study it appears that this disturbing phenomenon can at least partially be explained by small lymph node tumor infiltrates that are missed during standard histopathological analyses.

In accordance with our data there is an increasing body of evidence that immunohistochemical and molecular tumor cell detection identifies patients with poorer prognosis [8, 10–12]. It appears that the conventional pathologic LN assessment with H&E staining, as proposed by the College of American Pathologists, is not sufficient [32]. It is a logical consequence that a more in depth analysis of LN with the highest probability of harbouring ITC (the sentinel lymph nodes) would reflect more accurately the real tumor burden. Based on these considerations the SLN mapping procedure in colon cancer has been advocated by different research groups in Europe as well as North America [16, 18–20, 33]. In the present study all prospectively included patients were analyzed according to a standardized SLN protocol using immunohistochemistry to identify ITC. The present investigation clearly demonstrates a negative prognostic impact of ITC in SLN on disease-free survival. Furthermore, ITC was a poor prognostic factor for overall survival in risk adjusted Cox regression analyses.

Comparing data from different studies requires a uniform system of classifying small nodal tumor infiltrates. However, the use of an inconsistent nomenclature in many studies renders a comparison among published studies difficult [8, 10, 22]. The TNM staging system defines single tumor cells or tumor cell clusters ≤ 0.2 mm as ITC which are pathologically classified as pN0(i+) [24]. In our series, the prevalance of pN0(i+) patients was 31 % and confirms the average detection rate of most other studies using immunohistochemistry [8, 11, 12, 14, 18, 20].

As an indicator for surgical quality and pathological thoroughness a minimum of 12 resected LN are required for adequate colon cancer staging. An average of 28.5 ± 11.7 resected LN and 5.8 ± 3.4 collected SLN reflect an excellent quality of oncological resection and pathological dissection. This may also reflect the relatively low recurrence rate of 4.1 % of our patients after a median follow up of 4.6 years.

In this study we report on a collective of 74 stage I & II colon cancer patients. Most previously published studies did not differentiate between colon and rectal cancer [10, 16, 21]. However, staging, therapeutic regimens, prognosis and even recurrence patterns differ between colon and rectal cancer. We thus advocate that the two tumor types should be considered as different diseases and analysed separately.

We would like to acknowledge the limitations of the present investigation. First, this analysis is a cohort study and not a randomized controlled trial. However, for the research question at hand, it is simply not possible to perform a randomized trial. Second, potential bias due to unknown confounding cannot be completely excluded. And finally, the patient number was rather low, probably explaining the lack of a significant result for the association between ITC and overall survival in unadjusted risk analysis. However, the disadvantageous effect of ITC persisted after risk-adjustment both for overall and disease-free survival. Moreover, this study is among the largest to investigate the prognostic impact of ITC on disease-free and overall survival.

The current ASCO, NCCN and ESMO guidelines recommend adjuvant chemotherapy for stage II patients featuring high risk factors for tumor recurrence [3–5]. The patients in the presented trial did not receive adjuvant chemotherapy unless the above-mentioned criteria were present. The question if adjuvant treatment should be offered to the subgroup of ITC positive patients was beyond the scope of our study, however, it is likely that such patients do benefit from adjuvant chemotherapy.

## Conclusions

This study provides compelling evidence that ITC are an independent poor prognostic factor for disease-free and overall survival and pN0(i+) colon cancer should therefore be considered as a high risk factor. Hence, the identification of true node negative patients, which are cured by surgical resection alone, should be considered in future studies and guidelines.

## Abbreviations

LN: lymph node
SLN: sentinel lymph node
ITC: isolated tumor cells
H&E: hematoxylin-eosin
CK19: cytokeratin 19
ASCO: American society of clinical oncology
NCCN: National comprehensive cancer network
ESMO: European society for medical oncology
